# Correction to: Combining heterogeneous subgroups with graph-structured variable selection priors for Cox regression

**DOI:** 10.1186/s12859-021-04552-3

**Published:** 2022-01-11

**Authors:** Katrin Madjar, Manuela Zucknick, Katja Ickstadt, Jörg Rahnenführer

**Affiliations:** 1 Department of Statistics, TU Dortmund University, 44221 Dortmund, Germany; 2 Department of Biostatistics, Oslo Centre for Biostatistics and Epidemiology, University of Oslo, 0317 Oslo, Norway

## Correction to: BMC Bioinformatics (2021) 22:586 10.1186/s12859-021-04483-z

Following the publication of the original article [1], the authors identified a printing error in the indicator function. The correct indicator function is given below.

The incorrect indicator function:

$$\delta_{m} = {\mathbb{1}}\;\left( {T_{m} \le C_{m} } \right)$$

The correct indicator function:

$$\delta_{m} = 1\;\left( {T_{m} \le C_{m} } \right)$$

The original article [1] has been corrected.
