# Supplementary material for: Combining heterogeneous subgroups with graph-structured variable selection priors for Cox regression
Source: BMC Bioinformatics. 2021 Dec 11;22:586. doi: 10.1186/s12859-021-04483-z (PMC8665528; doi:10.1186/s12859-021-04483-z)
Supplement: Supplementary file 1 — Additional file 1: Additional supporting information for the results of the simulation studies and the case study. [file 12859_2021_4483_MOESM1_ESM.pdf]

Supporting information for "Combining heterogeneous subgroups with graph-structured variable selection priors for Cox regression"

by Katrin Madjar, Manuela Zucknick,
Katja Ickstadt and Jörg Rahnenführer

Supplementary Figures

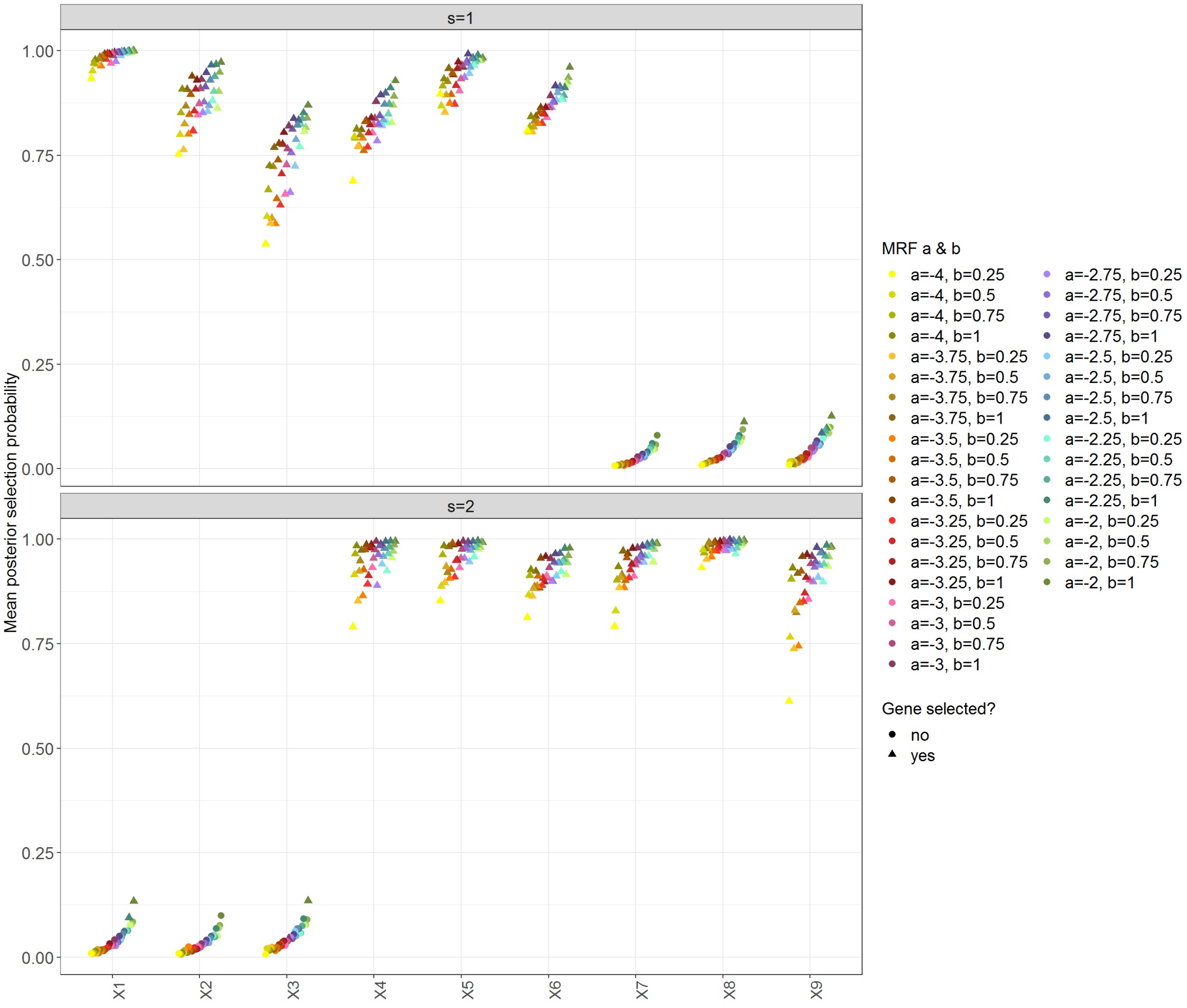

Fig 1: Mean posterior selection probabilities of the first nine genes in subgroups $s = 1, 2$ (averaged across five training sets) for all combinations of parameters a and b in the sensitivity analysis.

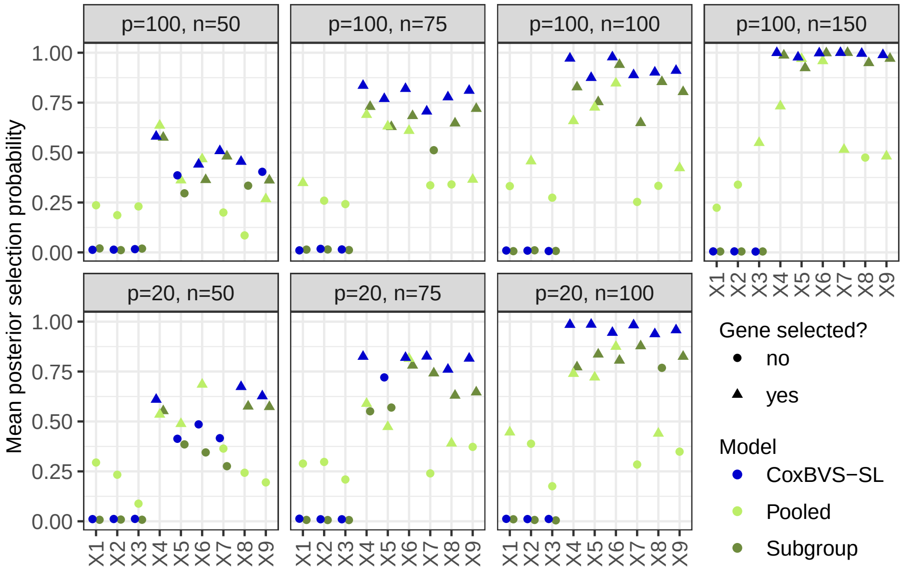

Fig 2: Mean posterior selection probabilities of the first nine genes in subgroup 2 (averaged across all training sets).

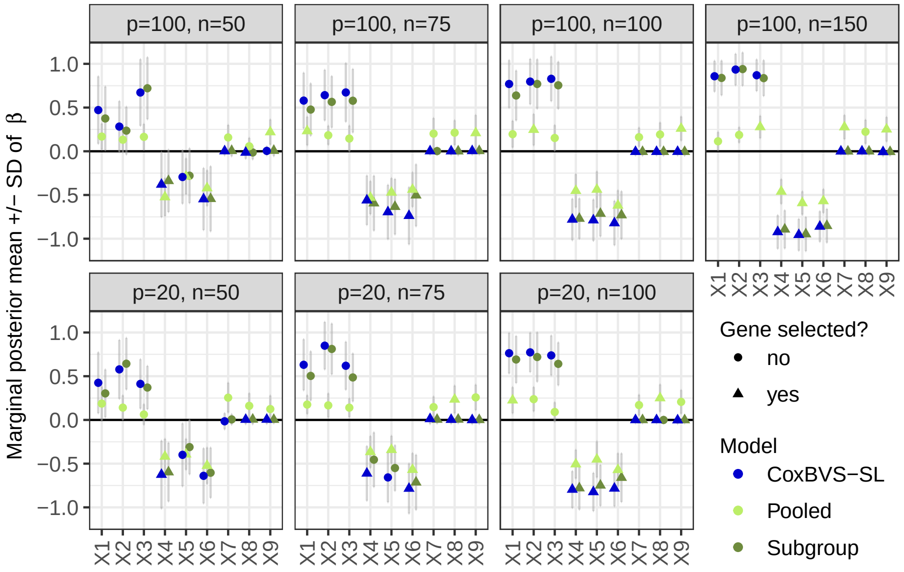

Fig 3: Marginal posterior means (independent of γ) and standard deviations (SD) of the regression coefficients of the first nine genes in subgroup 1 (averaged across all training sets).

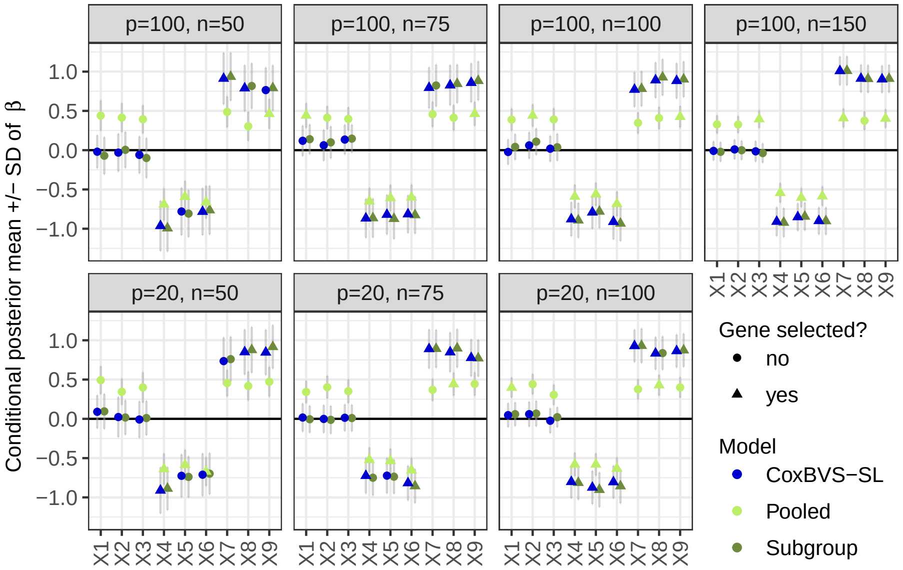

Fig 4: Conditional posterior means (conditional on $\gamma = 1$) and standard deviations (SD) of the regression coefficients of the first nine genes in subgroup 2 (averaged across all training sets).

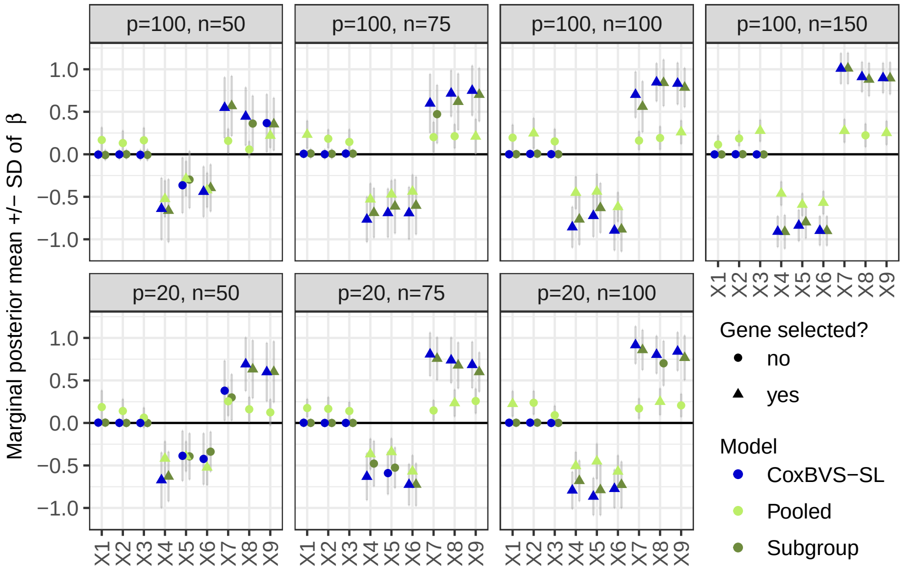

Fig 5: Marginal posterior means (independent of γ) and standard deviations (SD) of the regression coefficients of the first nine genes in subgroup 2 (averaged across all training sets).

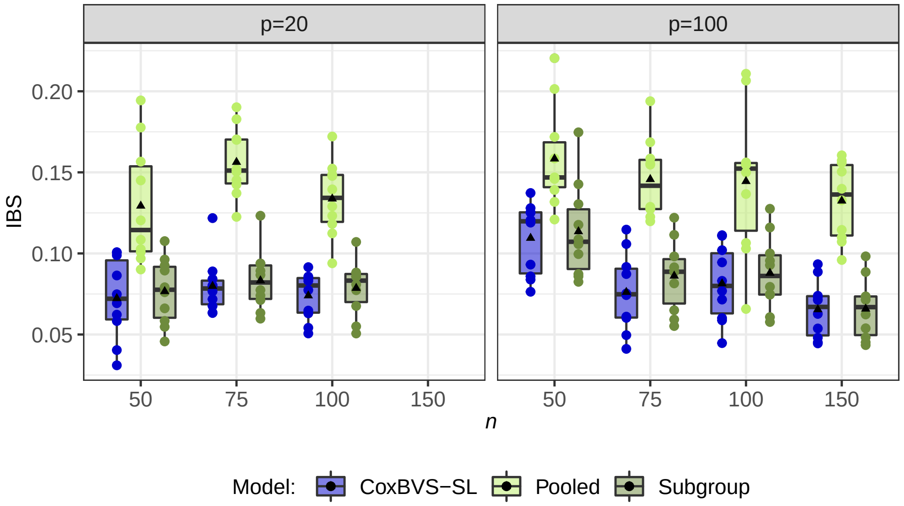

Fig 6: Integrated Brier Scores (IBS) across all ten test sets for subroup 1 (IBS based on the Bayesian Model Averaging). The black triangle within each boxplot represents the mean value.

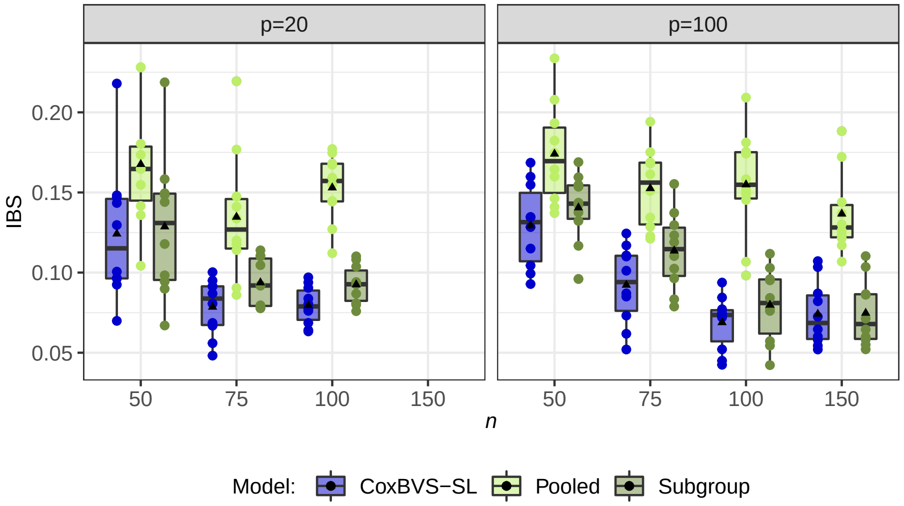

Fig 7: Integrated Brier Scores (IBS) across all ten test sets for subroup 2 (IBS based on the Median Probability Model). The black triangle within each boxplot represents the mean value.

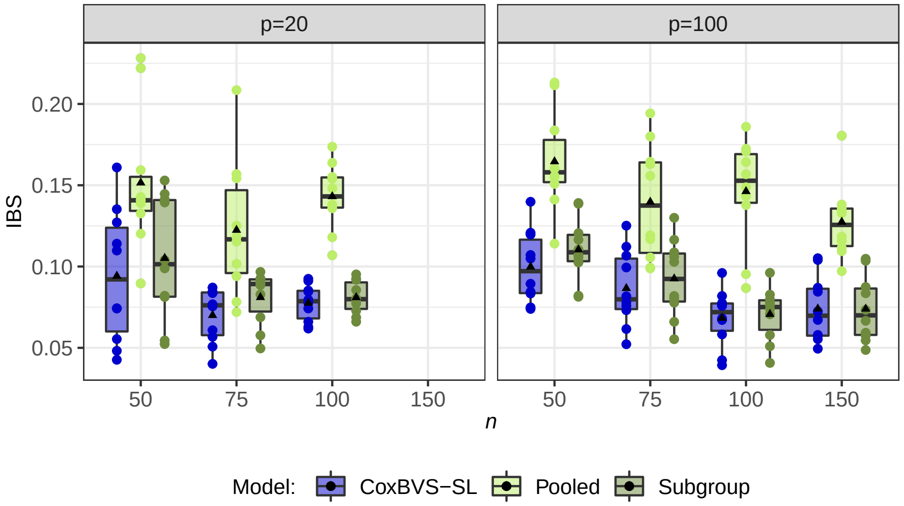

Fig 8: Integrated Brier Scores (IBS) across all ten test sets for subroup 2 (IBS based on the Bayesian Model Averaging). The black triangle within each boxplot represents the mean value.

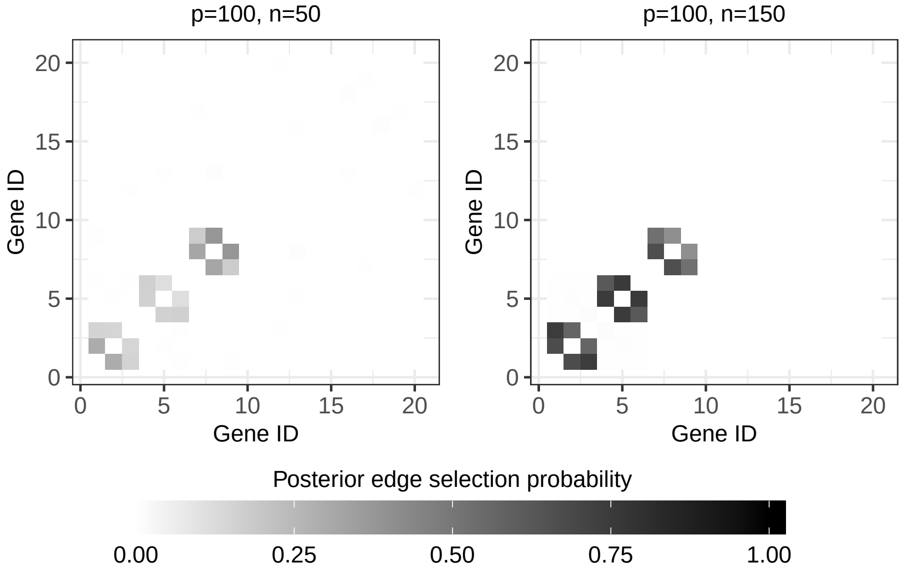

Fig 9: Marginal posterior edge selection probabilities of the first 20 genes in $\boldsymbol{G}_{11}$ (averaged across all training sets) for small and large n. Results for subgroup 2 are very similar.

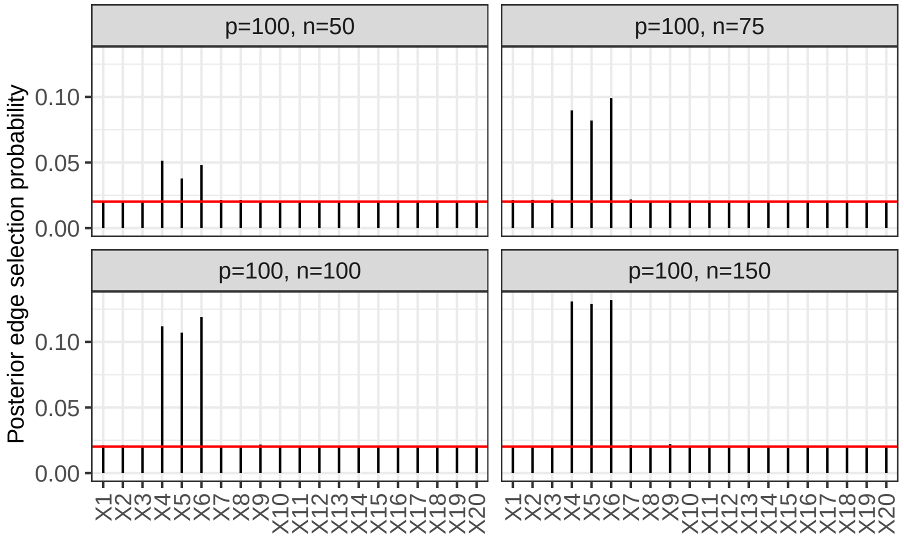

Fig 10: Marginal posterior edge selection probabilities of the first 20 genes in $\boldsymbol{G}_{12}$ (averaged across all training sets). The red line indicates the prior mean ($\pi_G = 2/(p-1) \approx 0.02$ for $p = 100$).

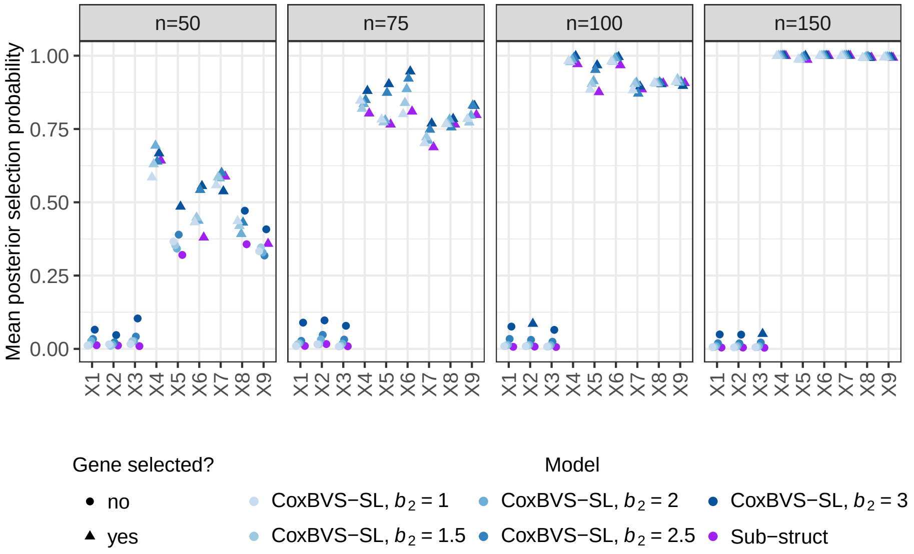

Fig 11: Mean posterior selection probabilities of the first nine genes in subgroup 2 (averaged across all training sets).

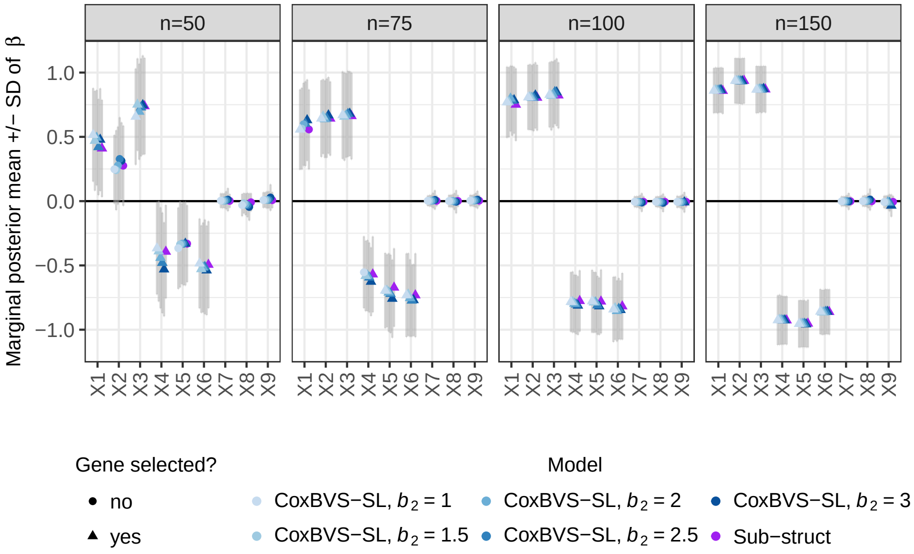

Fig 12: Marginal posterior means (independent of γ) and standard deviations (SD) of the regression coefficients of the first nine genes in subgroup 1 (averaged across all training sets).

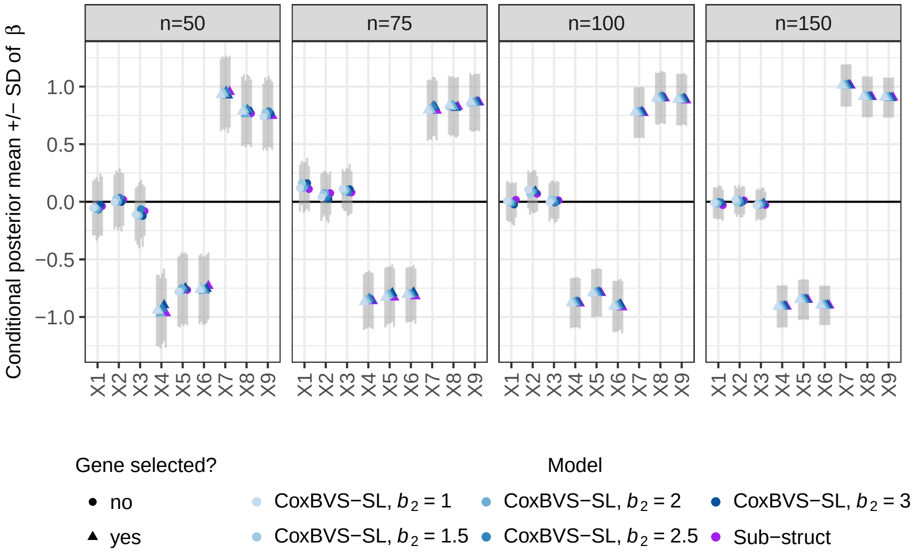

Fig 13: Conditional posterior means (conditional on $\gamma = 1$) and standard deviations (SD) of the regression coefficients of the first nine genes in subgroup 2 (averaged across all training sets).

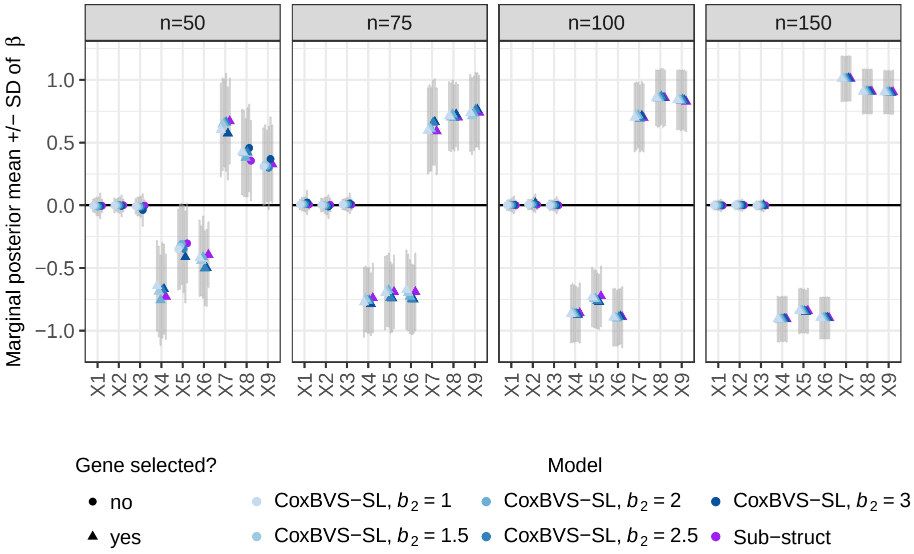

Fig 14: Marginal posterior means (independent of γ) and standard deviations (SD) of the regression coefficients of the first nine genes in subgroup 2 (averaged across all training sets).

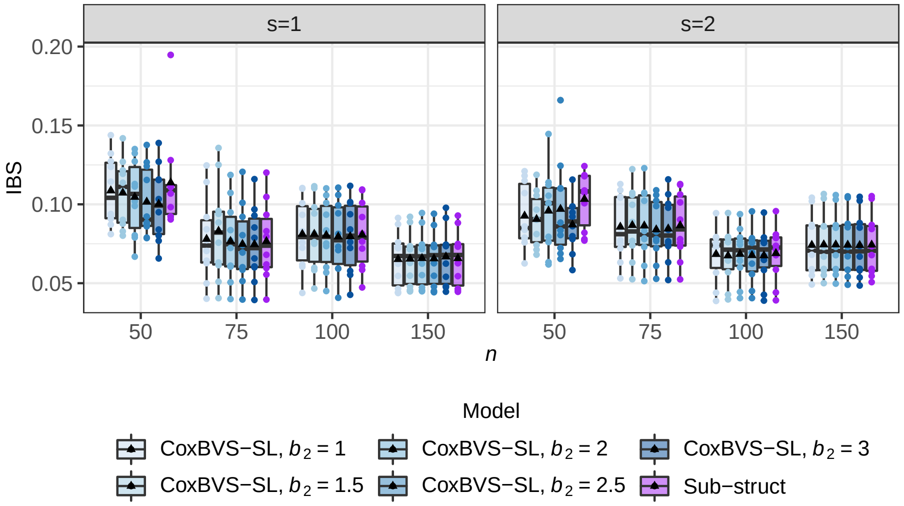

Fig 15: Integrated Brier Scores (IBS) across all ten test sets for subroup 1 (left) and 2 (right) (based on the Bayesian Model Averaging). The black triangle within each boxplot represents the mean value.

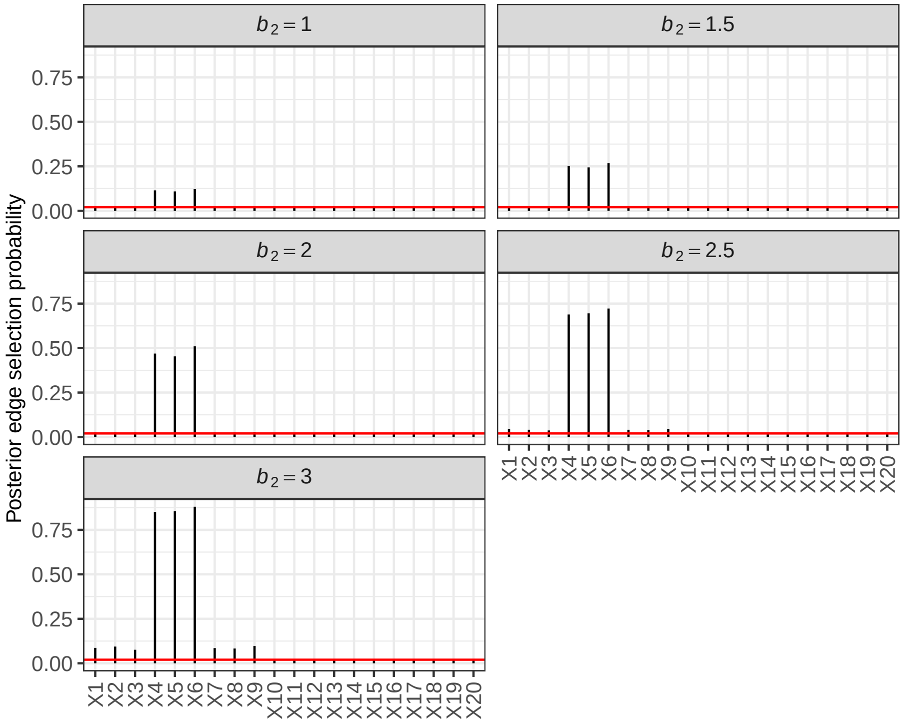

Fig 16: Marginal posterior edge selection probabilities (averaged across all training sets) of the first 20 genes in $\boldsymbol{G}_{12}$ and $n = p = 100$. The red line indicates the prior mean ($\pi_G = 2/(p-1) \approx 0.02$ for $p = 100$).

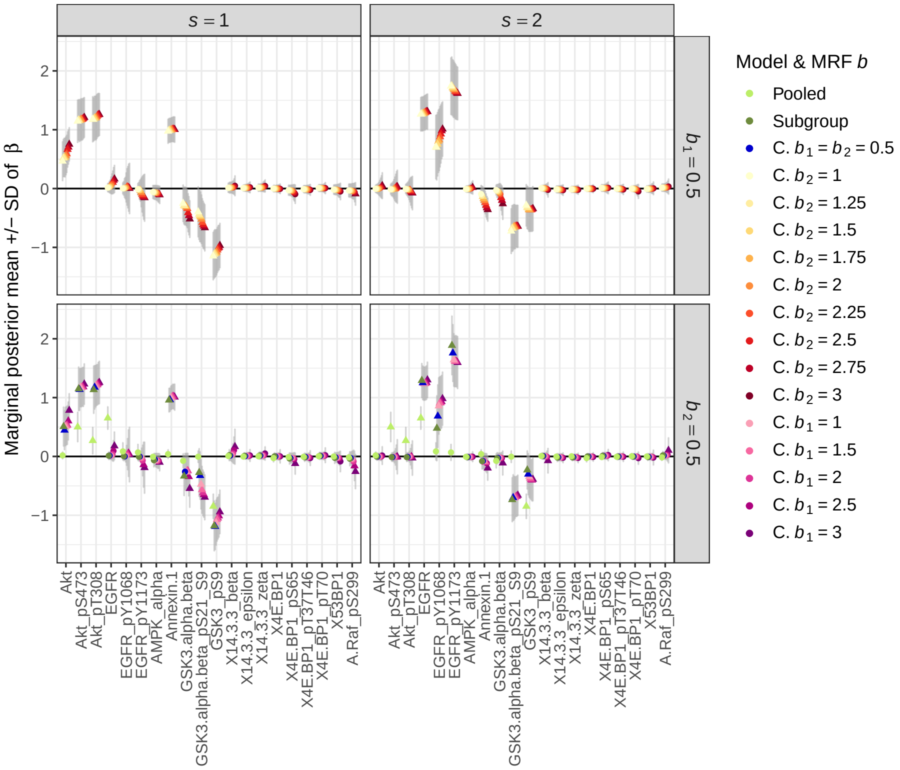

Fig 17: Marginal posterior means (independent of $\gamma = 1$) and standard deviations (SD) of the regression coefficients of all 20 proteins in both subgroups (averaged across all training sets). The different colors represent the models or parameter values of b_1 and b_2 in CoxBVS-SL (abbreviated by "C."). The plot symbol indicates whether a protein is selected (triangle) or not (circular point).

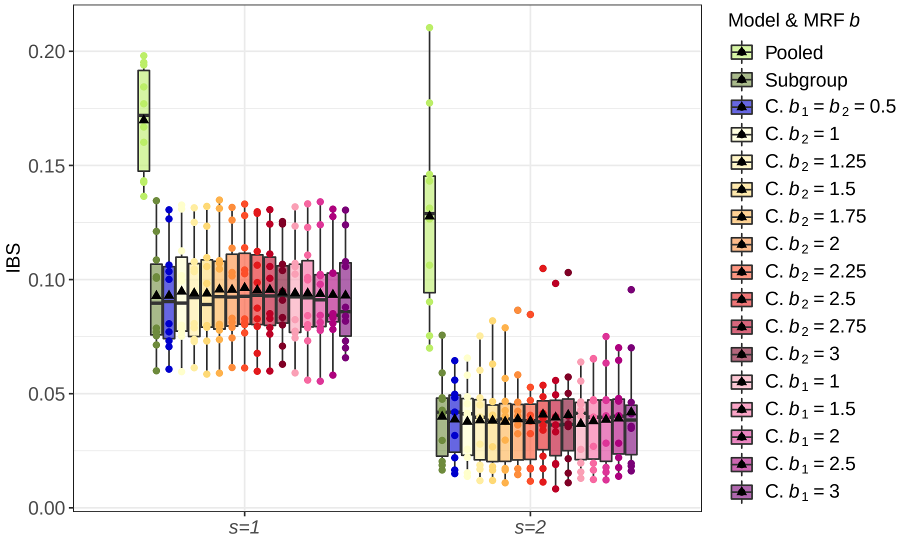

Fig 18: Integrated Brier Scores (IBS) across all ten test sets for both subroups (based on the Bayesian Model Averaging). CoxBVS-SL is abbreviated by ”C.”. The black triangle within each boxplot represents the mean value.

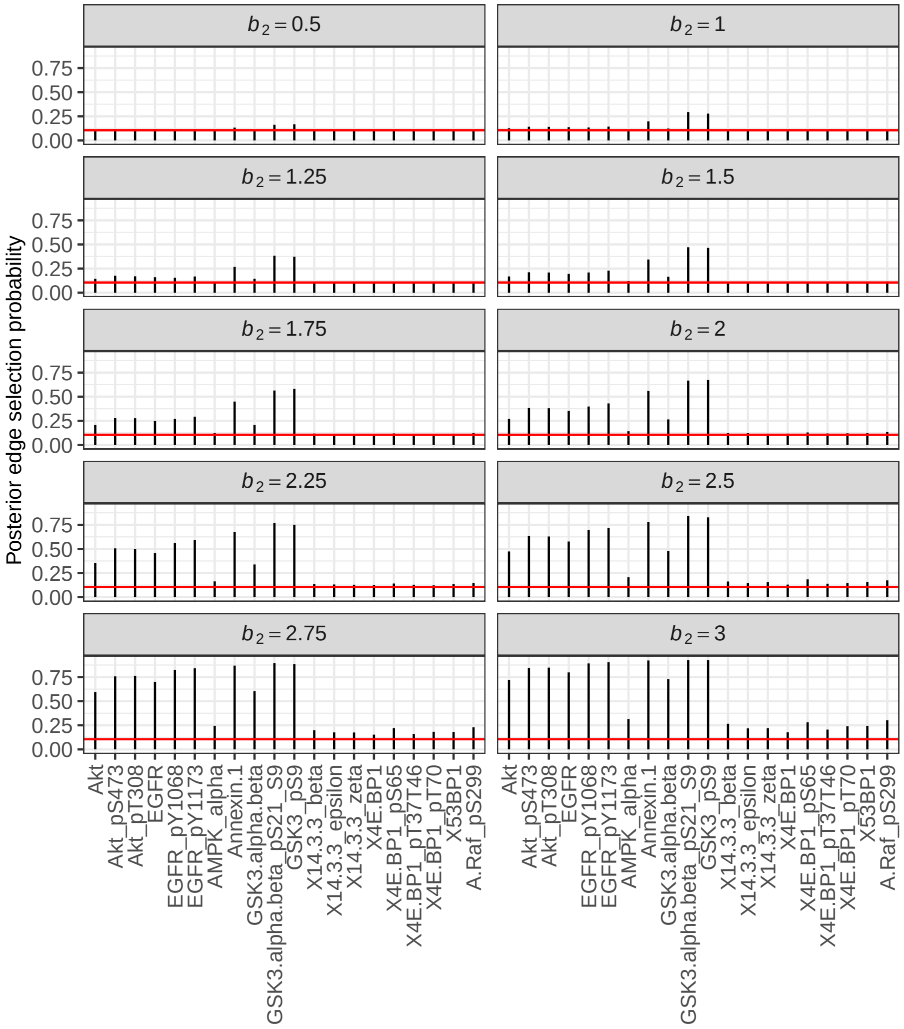

Fig 19: Mean marginal posterior edge selection probabilities for $\boldsymbol{G}_{12}$ (averaged across all training sets) in the CoxBVS-SL model with $b_1 = 0.5$. The red line indicates the prior mean ($\pi_G = 2/(p-1) \approx 0.11$ for $p = 20$).
